# 96-Channel on-chip reconfigurable optical add-drop multiplexer for multidimensional multiplexing systems

**DOI:** 10.1515/nanoph-2022-0319

**Published:** 2022-08-15

**Authors:** Weike Zhao, Yingying Peng, Xiaoping Cao, Shi Zhao, Ruoran Liu, Yihui Wei, Dajian Liu, Xiaolin Yi, Shangtong Han, Yuanjian Wan, Kang Li, Guangze Wu, Jian Wang, Yaocheng Shi, Daoxin Dai

**Affiliations:** State Key Laboratory for Modern Optical Instrumentation, Center for Optical & Electromagnetic Research, College of Optical Science and Engineering, International Research Center for Advanced Photonics, Zhejiang University, Zijingang Campus, Hangzhou 310058, China; Wuhan National Laboratory for Optoelectronics & School of Optical and Electronic Information, Huazhong University of Science and Technology, Wuhan, 430074, China; Optics Valley Laboratory, Wuhan 430074, Hubei, China

**Keywords:** add-drop, mode, multi-dimensional, multiplexing, silicon, wavelength

## Abstract

The multi-dimensional multiplexing technology is very promising for further increasing the link capacity of optical interconnects. A 96-channel silicon-based on-chip reconfigurable optical add-drop multiplexer (ROADM) is proposed and demonstrated for the first time to satisfy the demands in hybrid mode/polarization/wavelengthdivision-multiplexing systems. The present ROADM consists of a six-channel mode/polarization de-multiplexer, a 6 × 16 array of microring-resonator (MRR)-based wavelength-selective switches, and a six-channel mode/polarization multiplexer. With such a ROADM, one can add/drop optical signals to/from any channels of the multimode bus waveguide arbitrarily. For the designed and fabricated ROADM chip, there are more than 1000 elements integrated monolithically, including 96 MRRs, 576 waveguide crossings, 192 grating couplers, 96 micro-heaters, 112 pads, six polarization-splitter-rotators (PSRs), four asymmetric adiabatic couplers and four asymmetric directional couplers. For any channel added/dropped with the fabricated ROADM, the on-chip excess loss is about 5–20 dB, the inter-mode crosstalk is <−12 dB, and the inter-wavelength crosstalk is <−24 dB. The system experiments are demonstrated by using 10-GBaud quadrature phase shift keying (QPSK) signals, showing that the observed optical signal noise ratio (OSNR) power penalties induced by the ROADM are less than 2 dB at a BER of 3.8 × 10^−3^.

## 1 Introduction

The desire for high-capacity communication pushes the development of optical interconnects technology. Due to the Shannon limit and the erbium-doped fiber amplifier (EDFA) bandwidth limit, the widely used wavelength-division-multiplexing (WDM) technology hits its bottleneck [1]. The emerging mode-division-multiplexing (MDM) technology, which utilizes multiple modes as independent signal channels, provides a new dimension to further increase the traffic capacity [2–4]. Nowadays, the development of MDM has made considerable progress. Many key components have been developed for MDM systems, such as mode (DE)multiplexers (MUXs) [5, 6], multimode waveguide bends [7, 8], mode converters [9, 10], multimode waveguide crossings [11–13], mode filters [14], multimode power splitters [15, 16], etc. The system-level verification also proves the potential of the MDM systems [17, 18]. Currently, it is becoming more and more viable and attractive to develop a multi-dimensional multiplexing technology by combining *m* mode-channels, dual polarization-channels as well as *n* wavelength-channels together [19–21], because it increases the channel number greatly from *n* to 2 *mn*.

Consistent with its counterpart in WDM systems, there are some key devices/modules in hybrid MDM-WDM-PDM systems, including hybrid MUXs [22–25], hybrid switches [26–28], hybrid modulators [29], etc., which are usually constructed on the combination of high-performance mode/polarization/wavelength-manipulated elements, including mode MUX/DEMUXs [30–33], polarization rotators (PRs) [34–36], polarization beam splitters (PBSs) [37, 38], polarization splitter-rotators (PSRs) [39–41], micro-ring resonators (MRRs) [42, 43], arrayed-waveguide gratings (AWGs) [44–46], etc. Reconfigurable optical add-drop multiplexer (ROADM), which enables any channel to be switched and routed flexibly, is a key element in multiplexed optical communication systems [47–52]. In particular, recently ROADM for MDM systems has also attracted intensive attention [53–55]. In [53], a four-channel ROADM for MDM systems was demonstrated for the first time by integrating a pair of mode (DE)MUXs and four Mach–Zehnder switches (MZSs). Later, in [54] a ROADM for hybrid WDM-MDM systems was demonstrated for the first time by integrating a pair of mode (DE)MUXs and four microring-resonator-based wavelength-selective switches. However, it is still even challenging to develop a ROADM chip for multidimensional multiplexing systems because many wavelength-channels, a number of mode-channels, and dual polarization-channels are involved.

In this paper, we propose and demonstrate a 96-channel silicon-based on-chip ROADM for the first time to satisfy the demands in hybrid MDM-WDM-PDM systems. Here three modes, dual polarizations, and 16 wavelengths are involved. Accordingly, the present ROADM integrates a pair of six-channel hybrid MDM-PDM (DE)MUXs, and a 6 × 16 array of MRR-based wavelength-selective switches. In particular, the six-channel hybrid MDM-PDM (DE)MUXs are realized by introducing asymmetric adiabatic couplers for TE-polarization modes and asymmetric directional couplers (ADCs) for TM-polarization modes, regarding that there might be some notable polarization mode conversion due to the non-vertical sidewalls of the fabricated silicon photonic waveguides. For the designed and fabricated ROADM chip, there are more than 1000 elements integrated monolithically, including 96 MRRs, 576 waveguide crossings, 192 grating couplers, 96 micro-heaters, 112 pads, six polarization-splitter-rotators (PSRs), four asymmetric adiabatic couplers and four ADCs. With such a ROADM, one can add/drop optical signals to/from any channels of the multimode bus waveguide arbitrarily by switching the corresponding MRR-based optical switch. The fabricated ROADM chip shows on-chip excess losses (ELs) of 5–20 dB, inter-mode crosstalk of <−12 dB and inter-wavelength crosstalk of <−20 dB for adding any one of all 96 channels. The system experiments are demonstrated by using 10-GBaud quadrature phase shift keying (QPSK) signals, showing that the observed optical signal noise ratio (OSNR) power penalties induced by the ROADM are less than 2 dB at a BER of 3.8 × 10^−3^. The proposed scheme can be extended easily for even high capacity by adopting more mode- or wavelength-channels.

## 2 Principle and structural design

**Figure 1:**
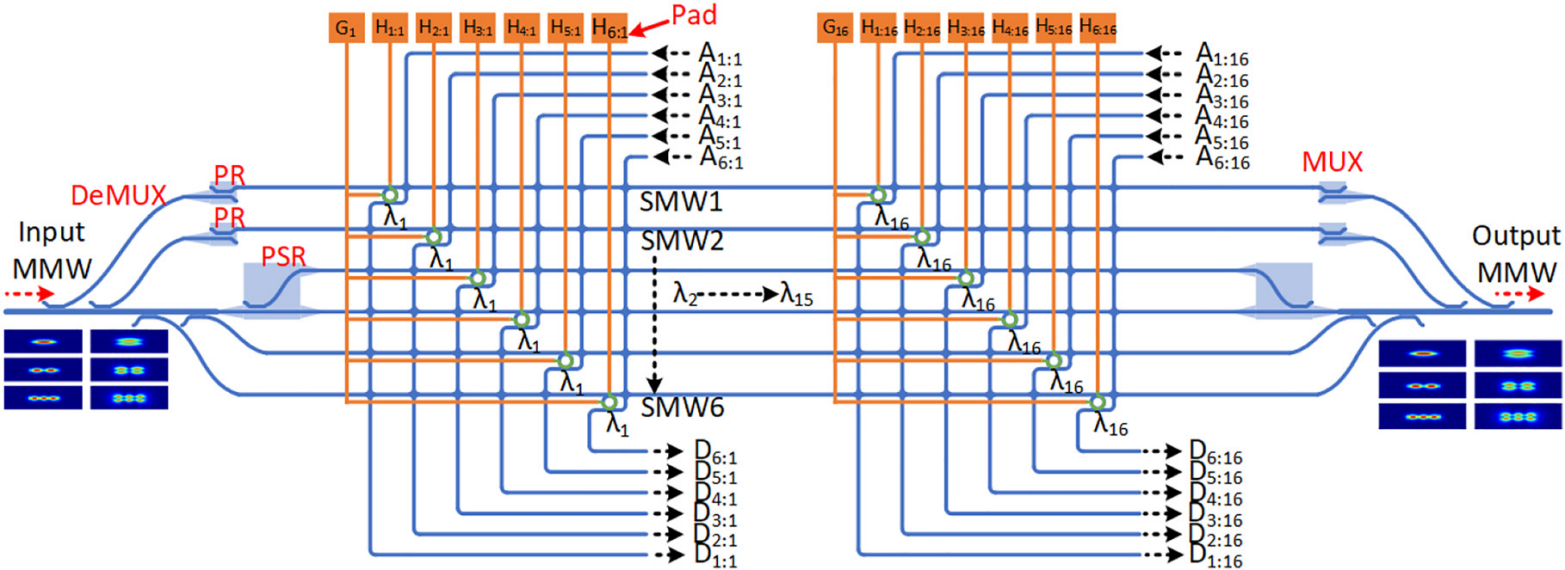
Configuration of the proposed ROADM for the hybrid MDM-PDM-WDM systems.

The present 96-channel ROADM for hybrid WDM-PDM-MDM systems is designed on the silicon-on-insulator (SOI) platform with a 220-nm-thick top-silicon layer and a 2 μm silica buffer layer. As shown in Figure 1, the ROADM consists of a pair of 6-channel hybrid MDM-PDM DEMUX, and a 6 × 16 array of MRR-based wavelength-selective switches. The 6-channel hybrid MDM-PDM DEMUX operates with three transverse electric (TE) modes (i.e., the TE_0_, TE_1,_ and TE_2_ modes) and three transverse magnetic (TM) modes (i.e., the TM_0_, TM_1_, and TM_2_ modes). The 6 × 16 MRR-based switches array has six rows corresponding to the six mode/polarization channels, and each row contains 16 cascaded MRR-based switches. The resonant wavelengths of these 16 cascaded MRRs increase from *λ*_1_ to *λ*_16_ with a channel spacing of Δ*λ*_ch_. Microheaters are used for the resonant wavelength tuning of each MRR-based switch, as shown in Figure 1, Pad *G*_*n*_ (*n* = 1–16) is the shared ground electrode of the *n*th wavelength channels, and *H*_*m:n*_ are the positive electrodes for *m*th mode and *n*th wavelength channels. Moreover, 6 × 96 waveguide crossings working on the principle of multimode interference are used for the crossing connection of the add/drop waveguides and the single-mode waveguides (SMWs) of the switches array [56]. For the hybrid MDM-PDM-WDM ROADM systems, data propagating along the multimode bus waveguide (MBW) are carried by 6 mode-, dual-polarization- and 16 wavelength-channels. When data arrives at the input MBW of the mode/polarization DEMUX, they are first demultiplexed into six TE_0_ modes by using the mode DEMUX together with the PRs/PSR, and then pass through the six SMWs of the 6 × 16 switches array, respectively. For each TE_0_ mode propagating through the SMW, the 16 wavelength-channels (*λ*_1_, … *λ*_16_) are dropped to port *D*_*m*:*n*_ (*n* = 1, … 16) or go through the SMW directly by switching the resonant wavelength of the corresponding MRR to/away from the target *λ*_*n*_. After passing through the switches array, these six TE_0_ modes from the SMWs are then multiplexed into the MBW by another mode MUX combined with PRs/PSRs. Similarly, when the *n*th wavelength-channel (*λ*_*n*_) is dropped to port *D*_*m*−*n*_, one can also add local signals carried by wavelength *λ*_*n*_ from port *A*_*m*:*n*_ to the same channel.

### 2.1 Mode (DE)multiplexer

**Figure 2:**
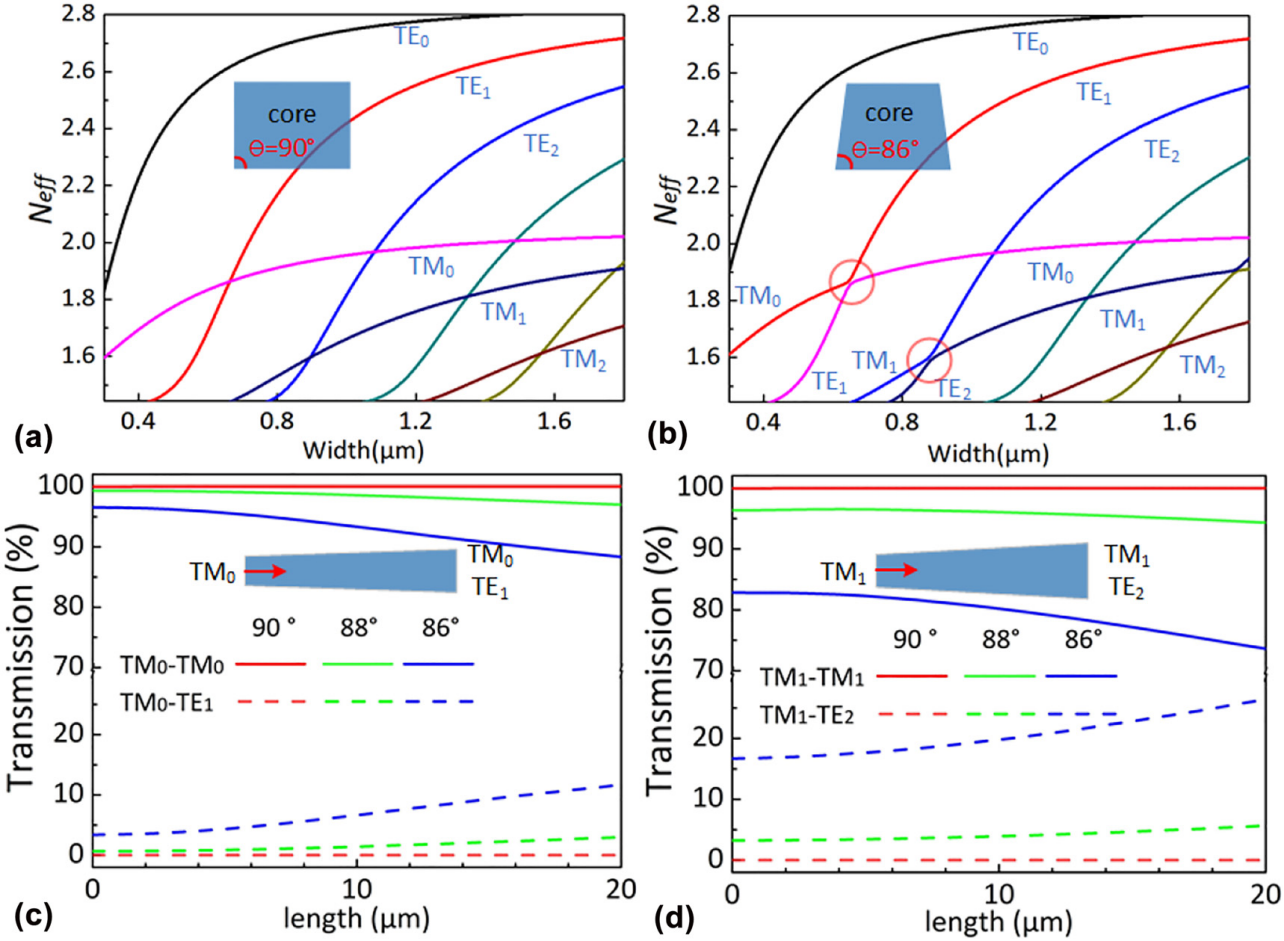
Calculated mode effective index (*N*_eff_) for the silicon photonic waveguides with 90° sidewalls (a) and 86° sidewalls (b). Calculated efficiency of the TM_0_-TM_0_/TE_1_ in a waveguide linearly tapered from 0.6 μm to 0.7 μm (c) and TM_1_-TM_1_/TE_2_ mode conversion in a waveguide linearly tapered from 0.82 μm to 0.92 μm (d), here different lengths (*L*_*t*_) and sidewall angles (*θ*) are chosen.

Various types of high-performance modes (DE)MUX on silicon have been proposed previously [30–33]. For these proposed modes (DE)MUX, as it might be noticed, the silicon photonic waveguides used are assumed to have a vertical sidewall. Unfortunately, the fabricated silicon photonic waveguides usually have angled sidewalls due to the imperfect dry etching process [57]. As pointed out in [57], there might be some mode hybridness and mode conversion when light propagates in a tapered waveguide with angled sidewalls. Figure 2(a) and (b) show the calculated effective indices *N*_eff_ of the guided modes in the 220-nm-thick silicon photonic waveguides with a sidewall angle of 90° and 86°, respectively. It can be seen that the TE_1_-TM_0_ and TE_2_-TM_1_ mode hybridness happens in the regions around the core width of 0.65 μm and 0.87 μm. When the waveguide is tapered around these two regions, the mode conversion happens, which might introduce some intermode crosstalk. This introduces lots of trouble for the design for the mode (DE)MUXes for which higher-order modes are involved. In particular, the TM_0_-TE_1_ mode conversions in a silicon photonic waveguide linearly tapered from 0.6 to 0.7 μm are analyzed, as shown in Figure 2(c). Meanwhile, Figure 2(d) shows the TM_1_-TE_2_ mode conversion in a silicon photonic waveguide linearly tapered from 0.82 μm to 0.92 μm. Here different lengths (*L*_*t*_) and sidewall angles (*θ*) are chosen. It shows that the mode conversion can be reduced by choosing nearly vertical sidewalls and a short taper. The crosstalk is less than −22 dB for the TM_0_-TE_1_ mode conversion when the angle *θ* > 88° and the taper length *L*_*t*_ < 6 μm. In contrast, when *θ* > 88° and *L*_*t*_ < 5 μm, the crosstalk is less than −15 dB for the TM_1_-TE_2_ mode conversion.

**Figure 3:**
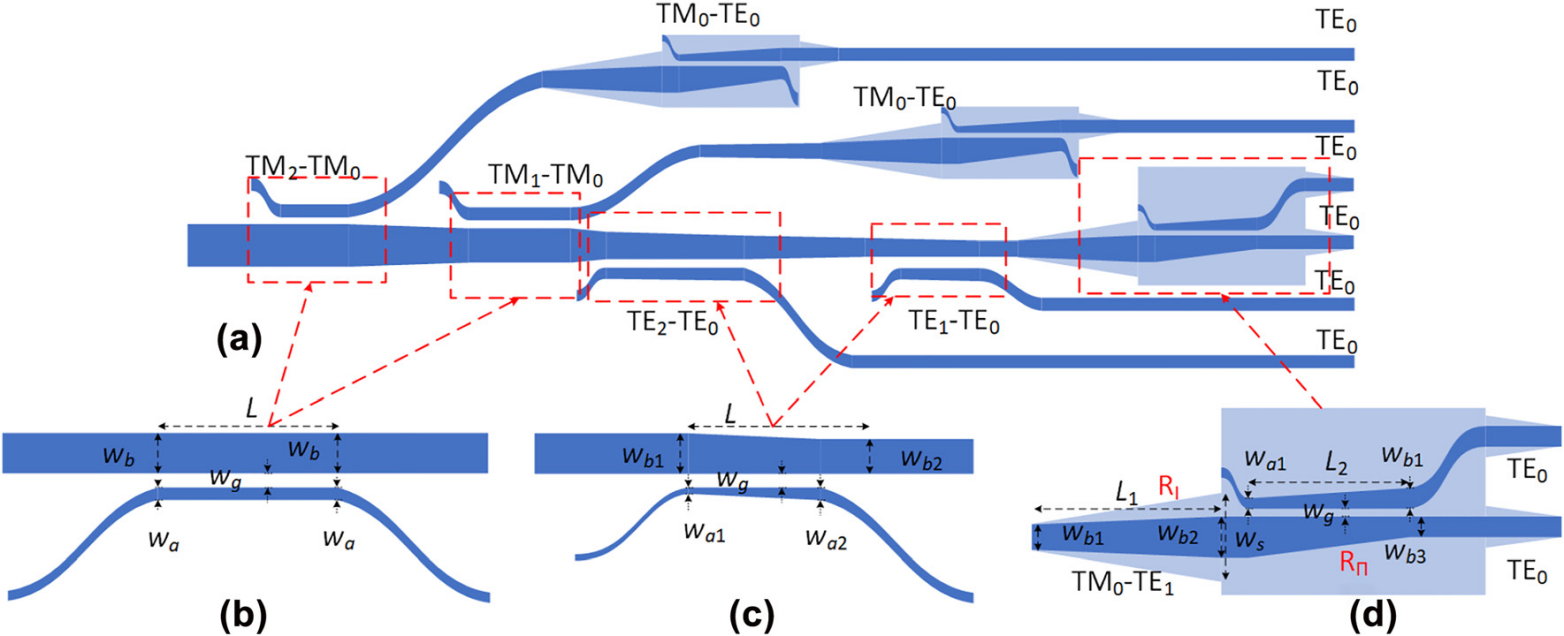
Schematic diagrams of the structures. (a) The mode/polarization (DE) multiplexer; (b) the ADC for TM modes; (c) the asymmetric-adiabatic-coupler for TE modes; (d) the PSR/PR.

**Figure 4:**
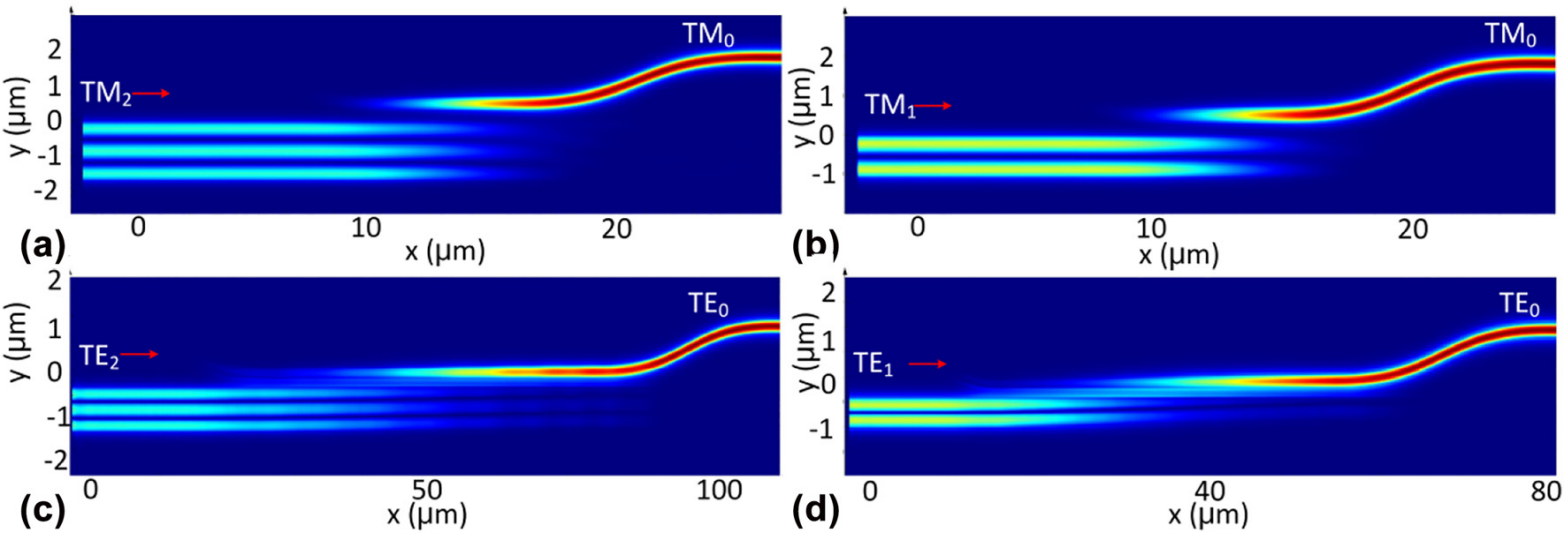
Light propagation of TM_2_(a), TM_1_(b), TE_2_(c), and TE_1_(d) in the corresponding coupler at 1550 nm wavelength.

**Figure 5:**
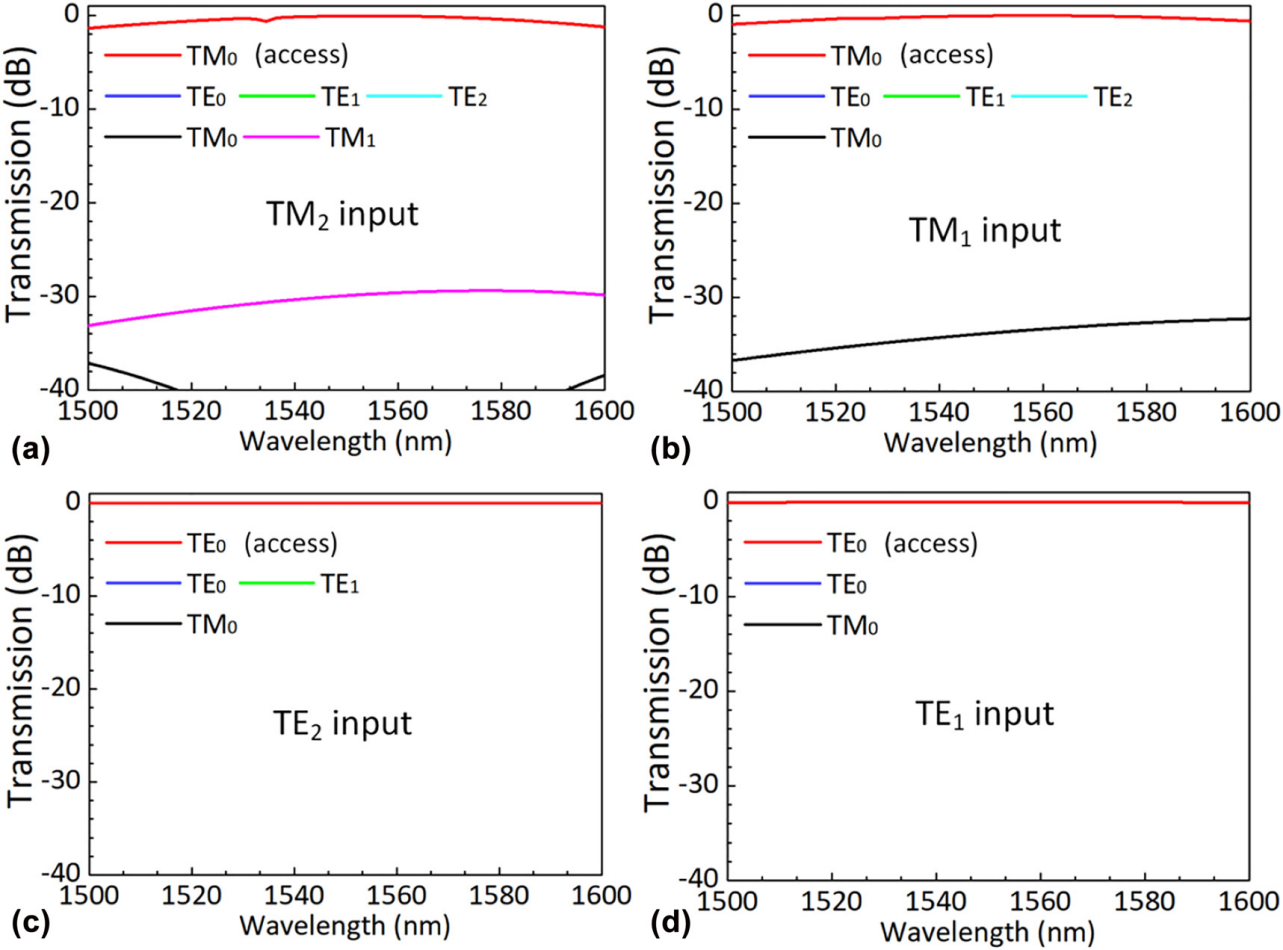
Calculated transmission of the launched TM_2_ (a), TM_1_ (b), TE_2_ (c), and TE_1_ (d) modes in the corresponding coupler in the wavelength range of 1500–1600 nm.

According to the analysis above, the adiabatic coupling region of (DE)MUX should be designed to exclude any mode hybridness region, while a short taper should be used when there is some mode hybridness for the waveguide whose width is varied from *w*_1_ to *w*_2_. Considering the mode effective index of TE modes are more sensitive to the waveguide width than that of TM modes, the mode/polarization (DE)MUXs are designed by combining the asymmetric adiabatic couplers for TE modes and ADCs for TM modes here. With such a hybrid scheme, one can achieve a mode MUX with a broad bandwidth and a large fabrication tolerance. Besides, one can also jump over the mode hybridization region with a nonadiabatic taper used in the uncoupled region to minimize the undesired intermode conversion. Figure 3(a) schematically shows the six-channel mode/polarization (DE)MUX, The TM_2_, TM_1_, TE_2_, and TE_1_ higher-order modes are decoupled to the TM_0_ and TE_0_ modes in four access waveguides successively, and the TM_0_ modes are further converted to the TE_0_ modes with the corresponding PRs. Moreover, the TE_0_ and TM_0_ modes that remained in the MBW are handled by a PSR, which converts the input TM_0_ mode to the TE_0_ mode at the output end [41]. Figure 3(b) shows the schematic diagram of the ADC-based mode DEMUX used for the TM modes. Here the wide-core width *w*_b_ and the narrow-core width *w*_a_ are chosen according to the phase-matching condition [30], and the coupling region length is chosen optimally. Figure 3(c) schematically shows the asymmetric-adiabatic-coupler-based mode DEMUX for the TE modes. The wide core is tapered from *w*_b1_ to *w*_b2_ with a length of *L*, and the narrow core is tapered from *w*_a1_ to *w*_a2_ correspondingly [33], while the gap between the wide core and narrow core is set as *w*_g_. The parameters chosen for the four couplers are listed in Table 1, and a 6-μm-long nonadiabatic taper is used to vary the waveguide width from 0.62 to 0.93 μm. Figure 4 (a–d) show the simulated light propagation for the TM_2_, TM_1_, TE_2_, and TE_1_ modes in the designed couplers when operating at the wavelength of 1550 nm. Finally all the modes are efficiently coupled to the TM_0_ or TE_0_ mode in the access waveguide. The transmissions of these four modes are shown in Figure 5(a–d). It can be seen that the ELs for the TM_2_, TM_1_, TE_2_, and TE_1_ modes are less than 1.3 dB, 0.95 dB, 0.02 dB, and 0.06 dB while the crosstalks are less than −30 dB over the wavelength range of 1500–1600 nm.

**Table 1:** Key parameters of couplers for four higher order modes.

Parameters	*w*_b1_ (μm)	*w*_b2_ (μm)	*w*_a1_ (μm)	*w*_a2_ (μm)	*w*_g_ (μm)	*L* (μm)
TM_2_	1.695		0.4		0.3	5.7
TM_1_	1.092		0.43		0.3	5
TE_2_	1.04	0.93	0.26	0.32	0.18	50
TE_1_	0.62	0.44	0.2	0.29	0.18	40

### 2.2 Polarization splitter rotators (PSRs) and polarization rotators (PRs)

**Figure 6:**
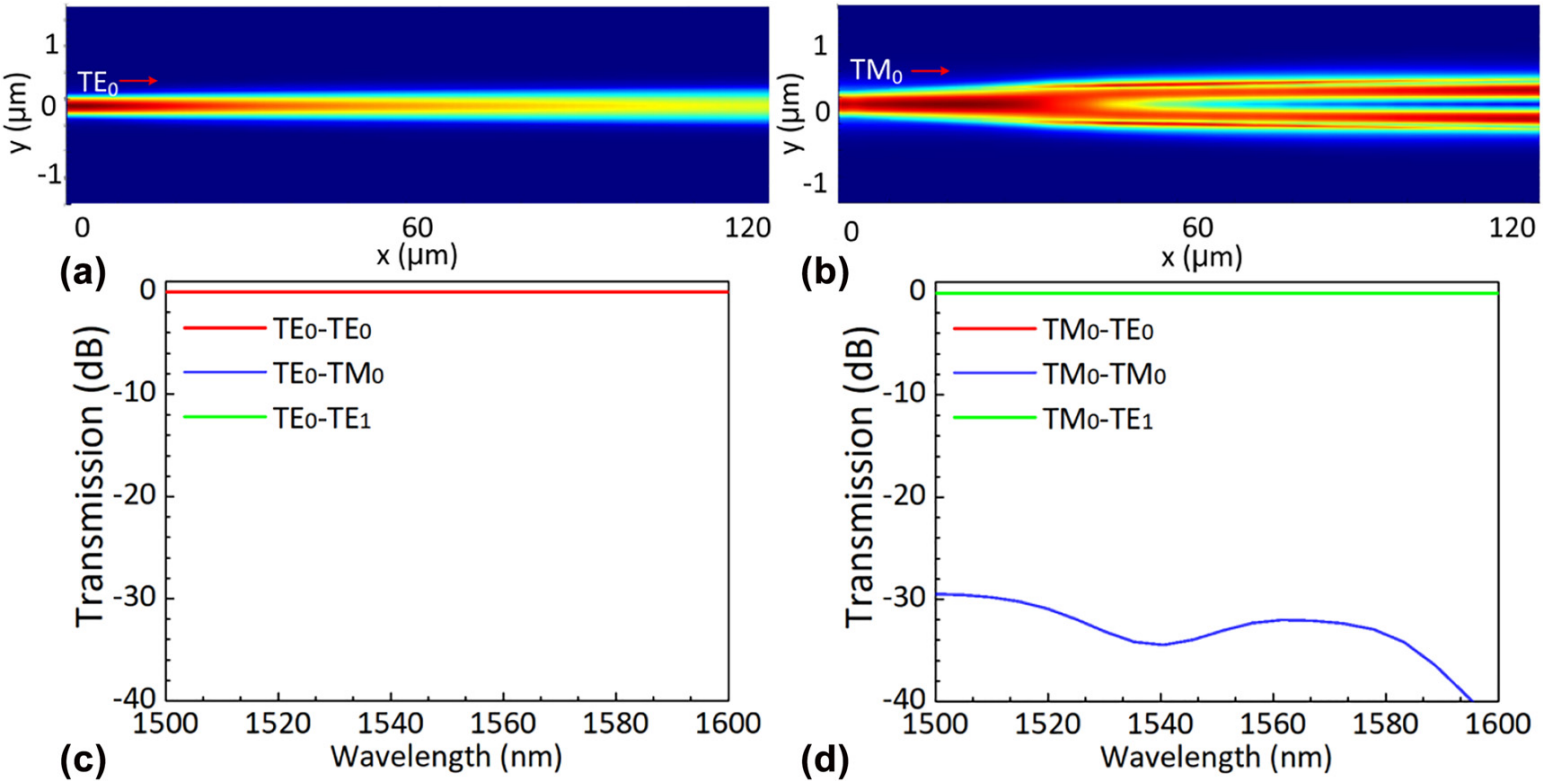
The simulated light propagation of the TE_0_ (a) and TE_1_ (b) modes in region R_I_. The calculated transmissions of the TE_0_ (c) and TE_1_ (d) modes in region R_I_.

**Figure 7:**
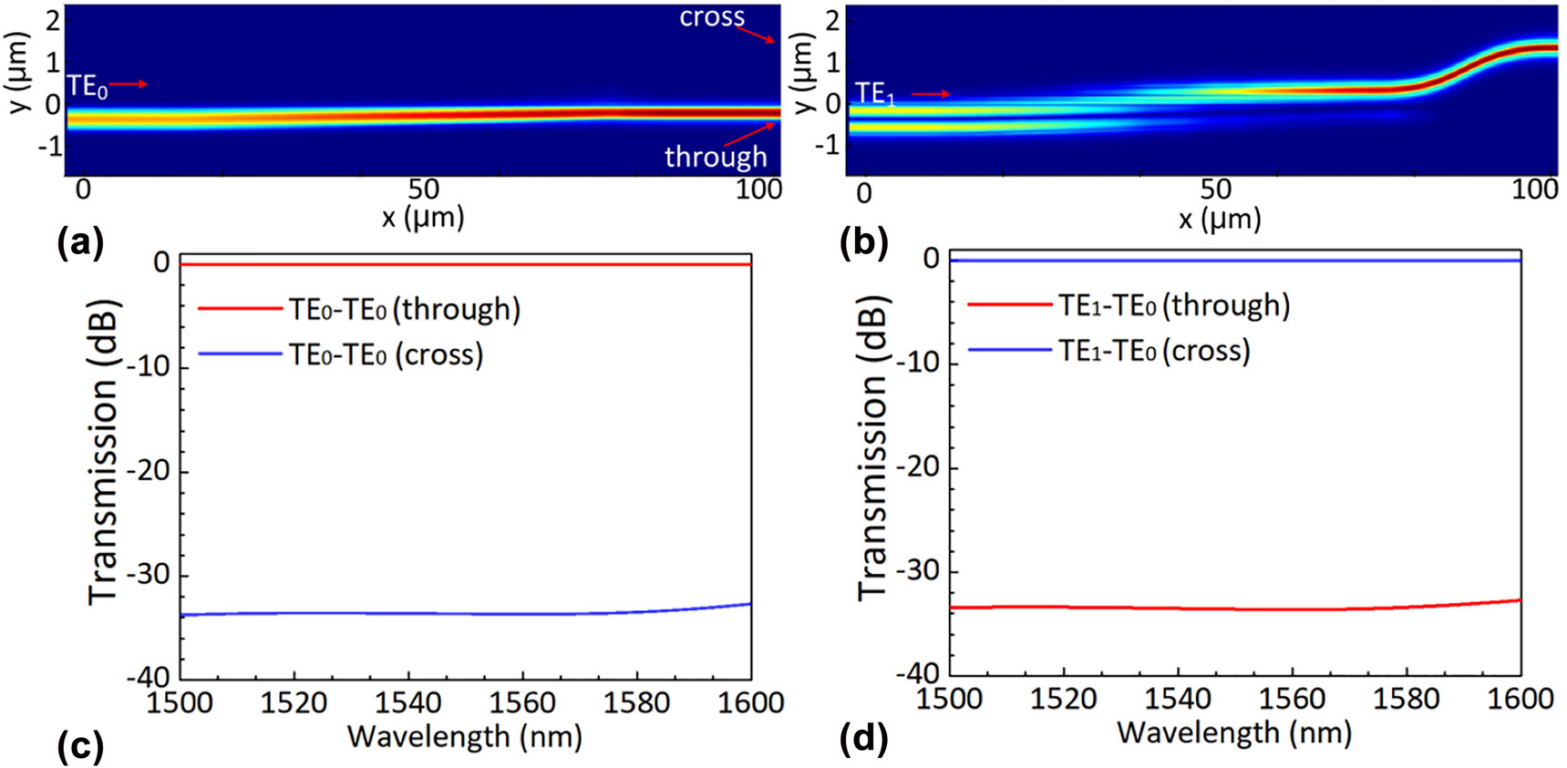
The simulated light propagation of the TE_0_ (a) and TE_1_ (b) modes in region R_II_. The calculated transmissions of the TE_0_ (c) and TE_1_ (d) modes in region R_II_.

The PSR works on the principle of mode hybridness of the ridge waveguide [41, 58], and the slab thickness *h*_s_ of the ridge waveguide is chosen as 70 nm to be compatible with the standard processes provided by the foundry. Figure 3(c) schematically shows the designed PSR, which contains two parts. One is the polarization rotation region (R_I_) and the other is the mode splitting region (R_II_). In region R_I_, the top ridge is linearly tapered from *w*_b1_ to *w*_b2_ while the bottom ridge is accordingly tapered from *w*_b1_ to *w*_s_ with a length of *L*_1_. As a result, the TM_0_ mode can be converted to the TE_1_ mode when it passes through region R_1_ due to the mode hybridness, while the TE_0_ mode passes through directly with an ultra-low loss. In region R_II_, the TE_1_ mode in the wide-core couples to the TE_0_ mode in the narrow core access waveguide by using an asymmetric adiabatic coupler, while the TE_0_ mode passes through this region without any notable coupling. The key parameters for PSR are summarized in Table 2 [59]. Figure 6(a) and (b) show the simulated light propagation in region R_I_ for the launched TE_0_ and TM_0_ modes. One can find that the TE_0_ mode passes through region R_I_ with negligible losses, while the TM_0_ mode is converted to the TE_1_ mode effectively. The corresponding transmission spectra over the wavelength range of 1500–1600 nm are shown in Figure 6(c) and (d). The TE_0_ mode has an EL less than 0.01 dB, and the TM_0_-TE_1_ mode conversion has an EL less than 0.08 dB as well as crosstalk less than −29 dB. Figure 7(a) and (b) shows the simulated light propagation of the TE_0_ and TE_1_ modes in region R_II_. It can be seen that the TE_0_ mode transmits through the bus waveguide directly and the TE_1_ mode couples to the TE_0_ mode in the narrow waveguide, as expected. From the calculated transmission spectra shown in Figure 7(c) and (d), one can find that the TE_0_ mode has an EL of ∼0.01 dB and the TE_1_ mode couples to the TE_0_ mode of the narrow waveguide with an EL less than 0.02 dB as well as crosstalk less than −34 dB. With the combination of regions R_I_ and R_II_, the TE_0_ and TM_0_ modes are finally converted to the TE_0_ modes at the two output waveguides. The polarization rotators (PRs) to rotate the TM_0_ mode to the TE_0_ mode are developed by idling the through port of the PSR.

**Table 2:** The key parameters of the PSR.

Parameters	*h*_*s*_	*w*_b1_	*w*_b2_	*w*_b3_	*w*_s_	*w*_a1_	*w*_b3_	*w*_g_	*L*_1_	*L*_2_
Value (μm)	0.07	0.42	0.72	0.42	2.0	0.18	0.3	0.18	130	60

**Table 3:** Summary of the reported silicon ROADM.

Year	Architecture	Channels	Transmission EL (dB)	Add/drop EL (dB)	Crosstalk (dB)
		(mode × polarization × wavelength)			
2013 [49]	Parent-sub MRR	4 × 1 × 1 = 4	2–4	∼2	−18 ∼ −20
2016 [50]	Cascaded MRRs	1 × 1 × 8 = 8	15–18	11–15	−8 ∼ −19
2016 [51]	Cascaded MRRs	1 × 1 × 12 = 12	3	0–2.5	−30
2016 [52]	AWG + MZS	1 × 1 × 8 = 8	∼15	∼7	−12 ∼ −18
2016 [54]	MUX + MZS	4 × 1 × 1 = 4	1–5	–	−15 ∼ −20
2017 [55]	MUX + MRR	4 × 1 × 1 = 4	–	2–5	∼−20
2020 [56]	MUX + MRR	3 × 1 × 1 = 3	∼7	∼10.7	∼−13.7
2020 [53]	ADC + Bragg	1 × 1 × 2 = 2	1–2.5	1–2.5	−12.8 ∼ −21.4
This work	MUX + PSR + MRR	3 × 2 × 16 = 96	∼20	∼18	<−12

### 2.3 MRR-based wavelength-selective switches

**Figure 8:**
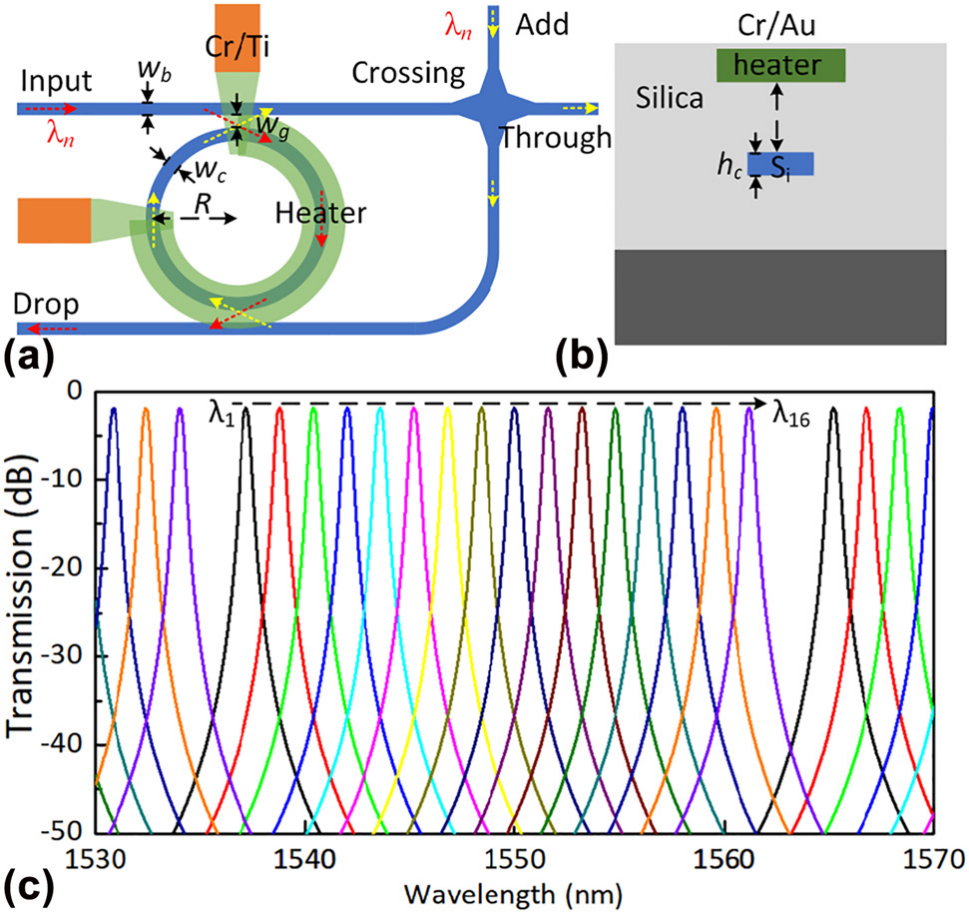
The MRR-based wavelength-selective switch. (a) The schematic diagram; (b) the cross-section of the MRR waveguide; (c) The calculated spectra for the drop port of the 16 MRRs in cascade.

**Figure 9:**
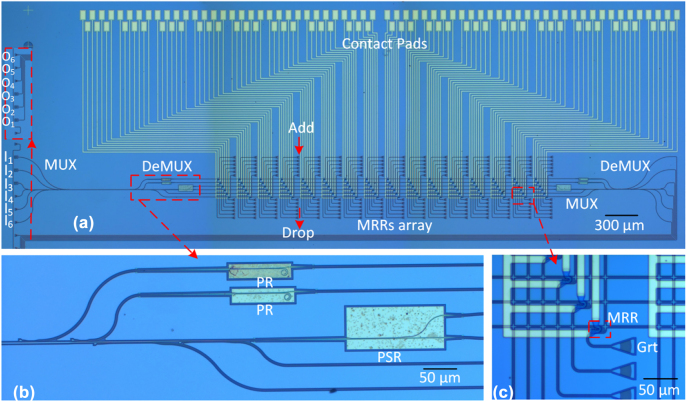
Microscopy images of the fabricated ROADM for hybrid WDM-MDM-PDM systems (a), the enlarged mode/polarization DEMUX (b), and the enlarged view for the MRR-based wavelength-selective optical switches (c).

Figure 9(a) shows the schematic configuration of MRR-based wavelength-selective optical switch. Two ports of the bus waveguide are used for the straight-forward transmission, and the two cross ports are used for adding and dropping light signals. The cross-section of the MRR waveguide is shown in Figure 9(b), where the metal heater is deposited on the silica upper-cladding along the MRR waveguide to tune the resonant wavelength. The widths of the bus waveguide and the core waveguide are chosen to *w*_b_ = 0.4 μm, *w*_c_ = 0.5 μm according to the phase-matching condition, and the waveguide gap is *w*_g_ = 0.19 μm. The MRRs are designed with a bending radius *R* varied by following the rule of *R* = 3.1771 + (*n* − 1)·0.0058 μm (where *n* = 1, … 16), in which way their resonant wavelengths increase with a channel spacing of 1.6 nm in the 1550 nm wavelength-band. Definitely a smaller bending radius ensures a larger free spectral range (FSR) for the MRR. Here we choose the bending radius *R* = 3.1771 μm, so that the FSR is as large as 27 nm to cover more than 16 channels. Figure 8(c) shows the calculated spectra for the drop port of the 16 MRRs in cascade.

## 3 Fabrication and results

The proposed ROADM was fabricated on an SOI wafer with a 220-nm-thick top-silicon layer and a 2-μm-thick BOX layer. The waveguide was patterned by using an E-beam lithography (EBL) process and formed by a dry-etching process, and then a 1.5-μm-thick SiO_2_ layer was deposited as the top cladding. Metal microheaters of Cr (20 nm)/Ti (200 nm) alloy were then fabricated by a lift-off process. Finally, a 200 nm thick silica was grown to protect the metal layer, and contact windows were opened upon the contact pad by a dry-etching process. Figure 9(a) shows the microscopy images for the fabricated 96-channels ROADM. A mode MUX and a mode DEMUX are connected to the input and output MBWs of the present ROADM, respectively, so that the mode channels can be characterized selectively. The TM-type and TE-type grating couplers are used for the fiber-chip coupling. For characterization convenience, the six input ports (i. e., *I*_1_–*I*_6_) and six output ports (i. e., *O*_1_–*O*_6_) are placed on the same side, in which way the package with a 12-channel fiber array can be carried out. The enlarged view of the six-channel mode/polarization (DE) MUX and the portion of the MRR-based wavelength-selective switches array are shown in Figure 9(b) and (c). The ROADM chip is bonded to a printed circuit board (PCB) and all 112 pads connected to the metal microheaters on the chip are wire bonded to the PCB for electrical control.

**Figure 10:**
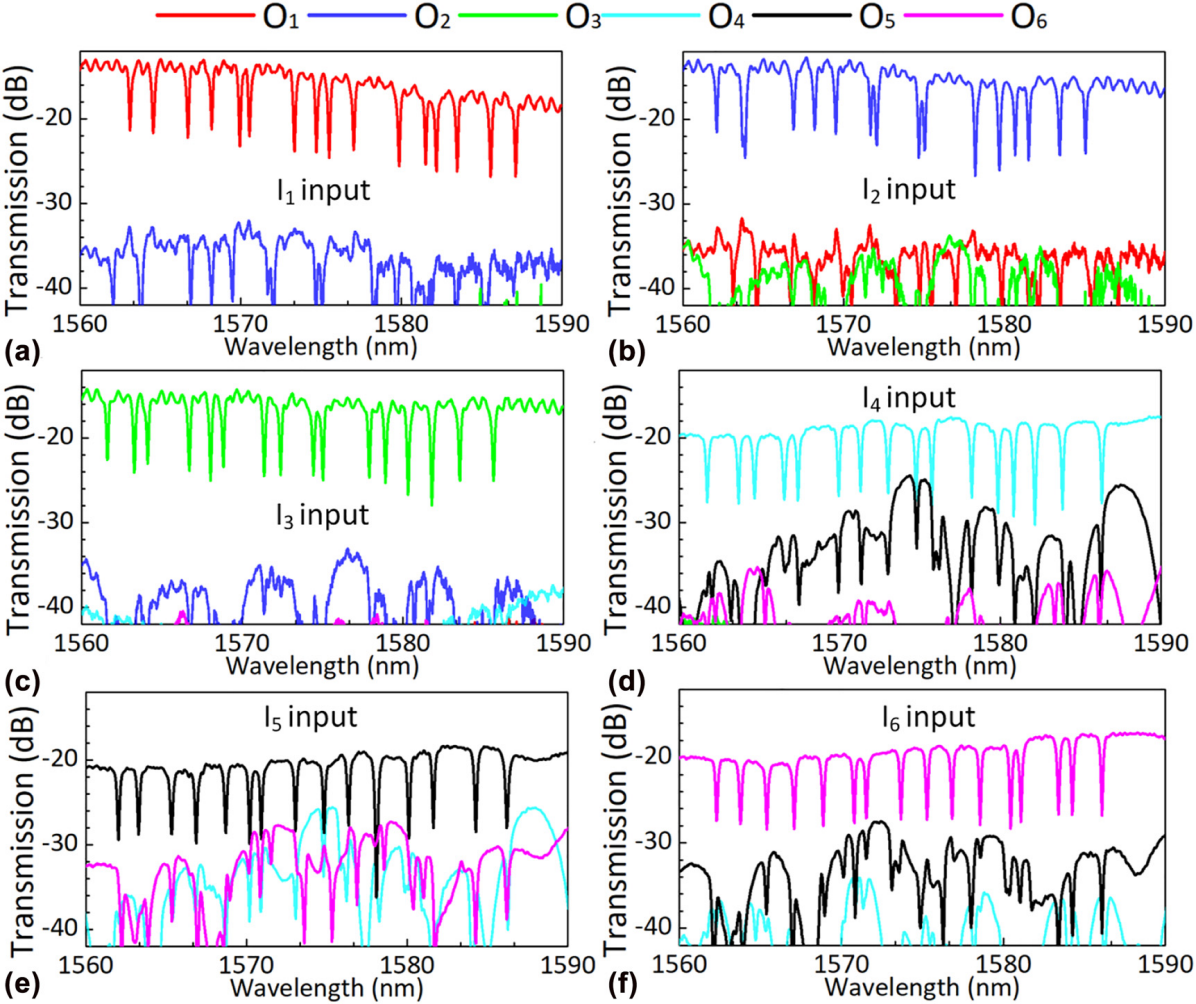
Measured transmission *T*_*ij*_ at the through port *O*_*j*_ (*j* = 1, 2, 3, 4, 5, 6) when light is inputted into (a) *I*_1_; (b) *I*_2_; (c) *I*_3_; (d) *I*_4_; (e) *I*_5_; (f) *I*_6_, respectively.

First the transmission spectra of the fabricated ROADM are measured. Light from a broadband source is launched into the input ports *I*_*i*_ (*i* = 1–6, corresponding to the TM_2_, TM_1_, TM_0_, TE_0_, TE_1,_ and TE_2_ mode channels) by a fiber array. Light from the six output ports *O*_*j*_ (*j* = 1–6) is then received with the same fiber array, which connects with an optical spectrum analyzer. Figure 10(a–f) show the measured transmission *T*_*ij*_ from port *I*_*i*_ to port *O*_*j*_. Here the transmission is normalized with respect to that for a straight waveguide fabricated on the same chip, when *i* = 1, 2, 3, 4, 5, and 6, respectively. The transmission *T*_*ij*_ is mainly dependent on the performance of the pairs of mode/polarization (de)multiplexers and the 6 × 16 MRR array.

When *j* = *i*, the transmission *T*_*ij*_ is ∼20 dB for TE modes and ∼15 dB for TM modes in the wavelength range of 1560–1590 nm, which includes several parts. First, the EL induced by the 0.7-cm-long loop-back waveguide is about 2 dB and 1.3 dB for the TE_0_ and TM_0_ modes, respectively, regarding that the propagation losses are ∼3.1 dB/cm and ∼1.8 dB/cm for the TE_0_ and TM_0_ modes, respectively. Second, the loss from the additional mode MUX/DEMUX placed at the input/output ends is about 3–4 dB. Accordingly, the EL for the optical signal transmitted to the through port of the fabricated ROADM has an EL of ∼14 dB and ∼10 dB for the TE and TM modes. Note that the waveguide crossing has an EL of ∼0.08 dB, thus the total EL due to the waveguide crossings involved is about 7.6 dB. Fortunately, the EL can be reduced further to ∼0.01 dB/crossing by optimizing the design of waveguide crossings with the particle swarm optimization (PSO) method [60].

When *j* ≠ *i*, the measured transmission *T*_*ij*_ gives the crosstalk from port *I*_*i*_ to port *O*_*j*_. It is shown that the inter-mode crosstalks (*T*_*ij*_ *–T*_*ii*_) are <−20 dB, −20 dB, −20 dB, −10 dB, −10 dB and −10 dB for the launched TM_2_, TM_1_, TM_0_, TE_0_, TE_1_ and TE_2_ mode, respectively. We also characterized the performances of a pair of mode MUXs connected back to back and a PSR fabricated on the same chip. It is shown that a pair of mode (de)multiplexers connected back to back have inter-mode crosstalk less than −20 dB for all six modes, while the PSR has low crosstalk less than −25 dB in the wavelength range of 1530–1600 nm. Moreover, there are some dips at specific wavelengths observed from the measured transmission of *T*_*ij*_, because the MRR-based switches drop the light signals carried by these wavelengths. The measured FSRs of the MRRs are ∼27 nm, which is consistent with the theoretical prediction.

**Figure 11:**
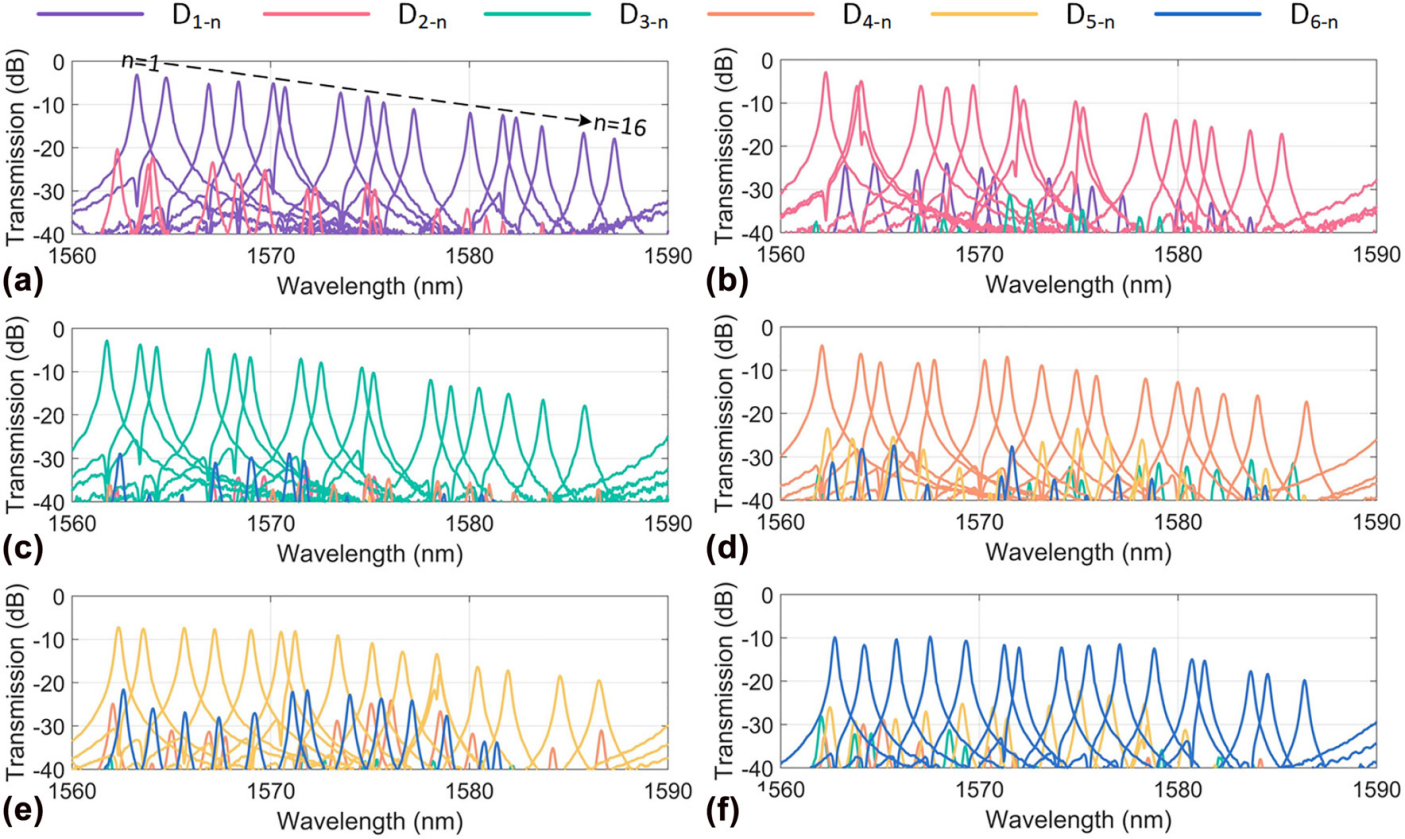
Measured transmissions *T*_*i−m−n*_ at drop port *D*_*m−n*_ (*m* = 1, 2, 3, 4, 5, 6) when light is input from port *I*_*i*_. (a) *i* = 1; (b) *i* = 3; (c) *i* = 3; (d) *i* = 4; (e) *i* = 5; (f) *i* = 6.

We also measured the spectra *T*_*i−m−n*_ at the drop port *D*_*m:n*_ (*m* = 1, … 6, *n* = 1, … 16) when light is input into port *I*_*i*_. Figure 11(a–f) show the measurement results in the wavelength range of 1560–1590 nm for the cases with port *I*_*i*_ (*i* = 1, 2, 3, 4, 5, 6), respectively. Take the case with port *I*_1_ as an example, which corresponds to the TM_2_ mode channel, as shown in Figure 11(a). When *m* = 1 and *i* = 1, The transmission *T*_1*−*1*−n*_ describes the signal propagation from the input port *I*_1_ to the drop port *D*_1:*n*_ (*n* = 1, … 16). The transmissions at the drop ports *D*_1:*n*_ are shown in Figure 11(a), and their resonance wavelengths are consistent with the dips of the transmission of *T*_11_ shown in Figure 10(a). The crosstalk to the other ports *D*_*m*:*n*_ (*m* ≠ 1) is also shown in Figure 11(a). The inter-mode crosstalk can be evaluated by (*T*_1*−m−n*_-*T*_1*−*1*−n*_) for a fixed wavelength channel *λ*_*n*_. It is shown that the inter-mode crosstalks are less than −20 dB, −20 dB, −20 dB, −15 dB, −13 dB, and −12 dB for the TM_2_, TM_1_, TM_0_, TE_0_, TE_1,_ and TE_2_ mode-channels, respectively. The fabrication error of the mode (DE)MUX induces the increased crosstalk for the TE_2_ and TE_1_ mode channels. It can be seen that the peak power of *T*_*i−i−n*_ becomes lower as *n* increases, because more MRRs and waveguide crossings are involved. As it might be noticed from Figure 11(a)–(f), for mode MUXes used here, the intermode crosstalk is usually low and much more wavelength-dependent than the excess loss. As a result, when the *i*th mode channel is launched, the envelope of the transmissions *T*_*i−m−n*_ (*i* ≠ *m*) at the drop ports *D*_*m−n*_ (*n* = 1, … 16) are fluctuant randomly. When injecting optical signals into the add port *A*_*m−n*_, one can add the data to the arbitrary mode- and wavelength-channel in the MBW by using the specific MRR-based wavelength-selective switch.

**Figure 12:**
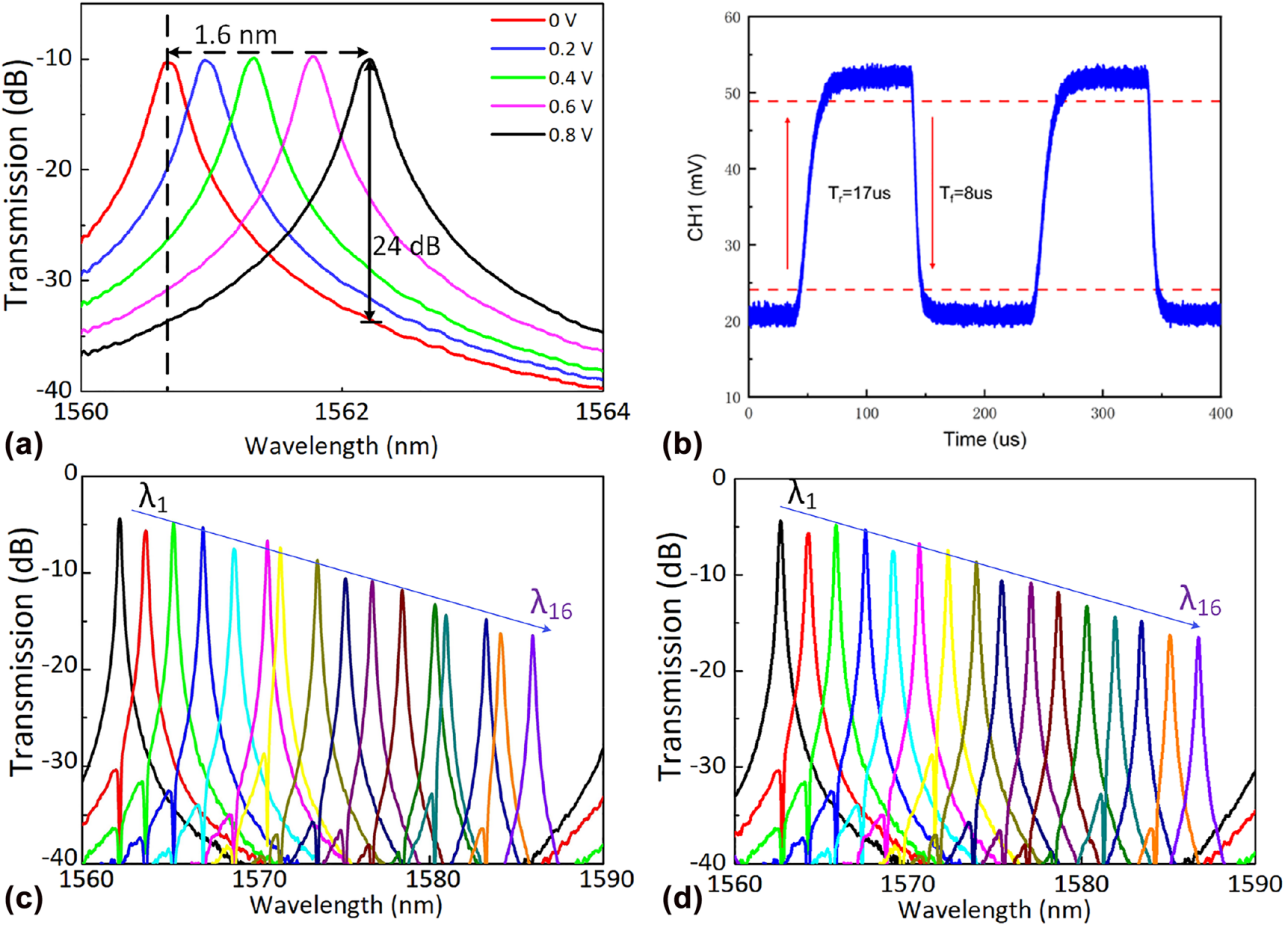
Measured transmissions (a) and switching responses (b) of the MRR-based wavelength-selective switch operating with different heating voltages. The measured drop spectra of the 16 MRR-based wavelength-selective switch without (c) and with (d) heater tuning.

Although the channel spacing of the 16 wavelength channels is not uniform due to the random fabrication deviations of MRRs, one can obtain uniform drop spectra by finely tuning the microheater of the MRR-based switch to be aligned with the grids of WDM systems. Figure 12(a) shows the tuning characteristics of an MRR-based wavelength selective switch. The resonant wavelength of the MRR-based switch has a 1.6 nm red-shift when the voltage applied on the heater increases from 0 to 0.8 V (i.e., ∼4.5 mW), which is sufficient to achieve the wavelength-selective adding/dropping. Figure 12(b) shows the switching response of the MRR-based switch, which has a switching rise-time of 17 μs and a drop-time of 8 μs. Here we take the transmission *T*_1*−*1*−n*_ (*n* = 1, … 16) as an example to show the tunability of the MRR-based switch. Figure 12(c) and (d) shows the measured drop spectra of the 16 MRRs in cascade without and with thermal tuning, respectively. The channel spacing between these MRRs is tuned to be 1.6 nm by applying appropriate power for heating. When the resonant wavelength of the MRR-based switch is aligned to the target wavelength channel *λ*_*n*_, the signal carried by this wavelength can be dropped from or added to the SMW. However, when the resonant wavelength of MRRs is aligned to *λ*_*n*_ + 0.5 Δ*λ*_ch_, which locates at the central position between two adjacent channels, the optical signal carried by the channel *λ*_*n*_ passes through the switches array directly without affecting the other wavelength channels almost. Figure 12(a) shows the 0.8 nm and 1.6 nm inter-wavelength crosstalk of the fabricated MRRs are less than −19 dB and −24 dB. As a result, one can selectively add/drop any mode- and wavelength-channel with low crosstalk by tuning the heating power.

**Figure 13:**
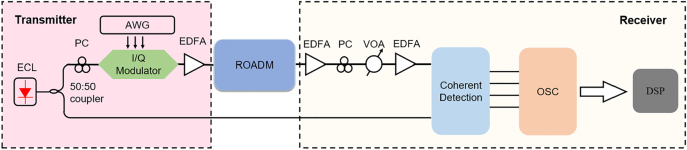
Experiment setup for the system transmission characterization with an external cavity laser (ECL), a 3-dB coupler, two polarization controllers (PCs), an arbitrary waveform generator (AWG), an I/Q modulator (Mod.), three erbium-doped fiber amplifiers (EDFA), a variable optical attenuator (VOA), a coherent detection receiver, an oscilloscope (OSC), and an offline digital signal processor (DSP).

We further characterized the system transmission performances of the present on-chip ROADM, where 10-GBaud quadrature phase shift keying (QPSK) signals are used and the configuration of the experimental setup is shown in Figure 13. For the transmitter, the optical carrier from an external cavity laser (ECL) at a specific wavelength was divided into two paths, one for generating the signal and the other for the local oscillator (LO). The QPSK signals are generated by the I/Q modulator driven by an arbitrary waveform generator (AWG) operated at 10-Gbaud. Then, the optical signals were amplified by an erbium-doped fiber amplifier (EDFA) and coupled into the fabricated ROADM chip by the grating couplers. After passing through the ROADM chip, the optical signals were coupled out of the chip and sent into the receiver, where the received QPSK signals and LO are mixed for coherent detection. Finally, the bit-error-rate (BER) performance was evaluated by digital offline processing after recording the received waveforms with a real-time sampling oscilloscope (OSC) operating at 80 GSa/s.

**Figure 14:**
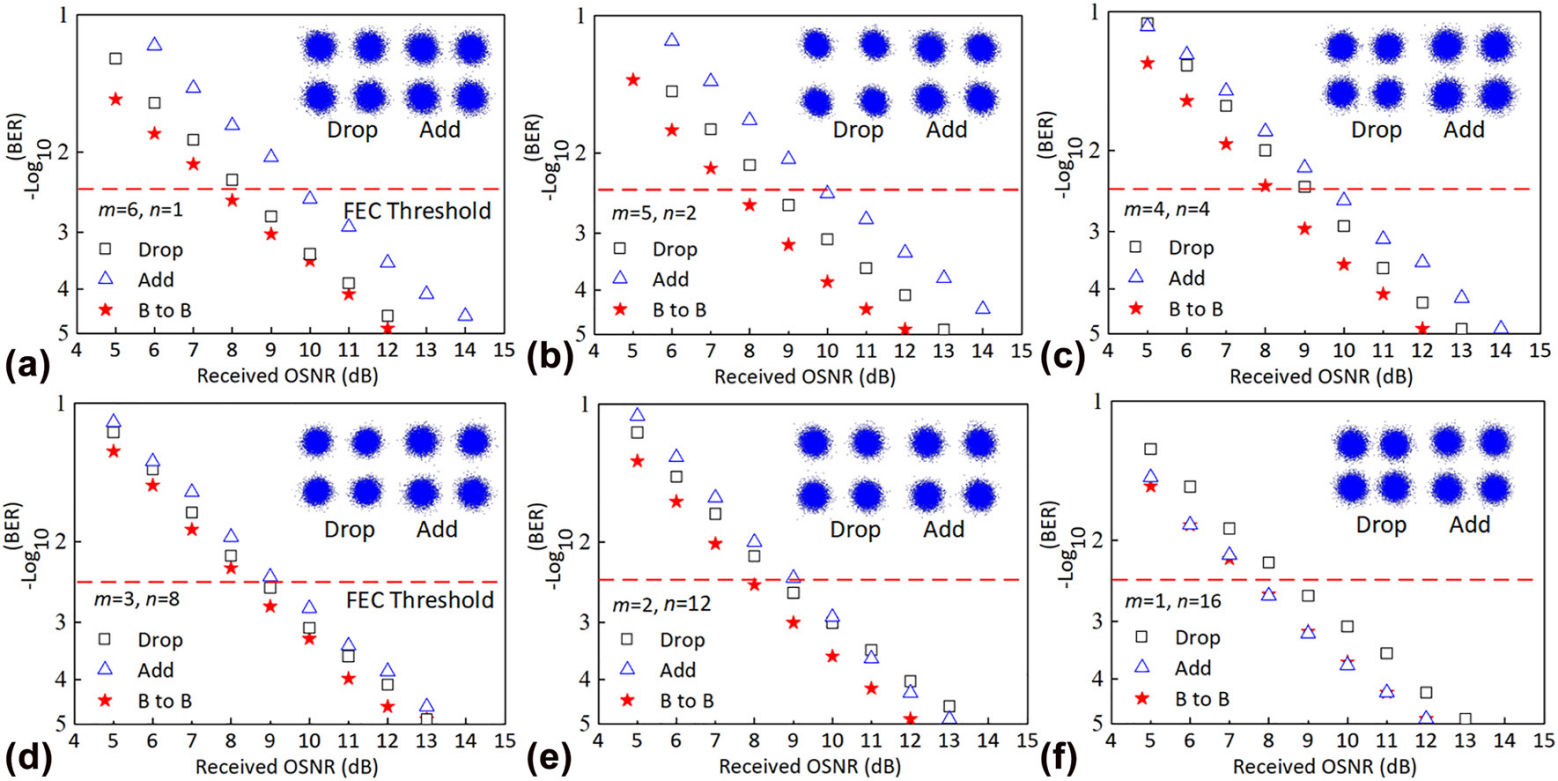
Measured BER performances of ROADM for *m*th modes and *n*th wavelengths (C_*m:n*_), (a) *m* = 6, *n* = 1; (b) *m* = 5, *n* = 2; (c) *m* = 4, *n* = 4; (d) *m* = 3, *n* = 8; (e) *m* = 2, *n* = 12; (f) *m* = 1, *n* = 16. Insets are constellations of QPSK for the dropping and adding transmissions.

**Figure 15:**
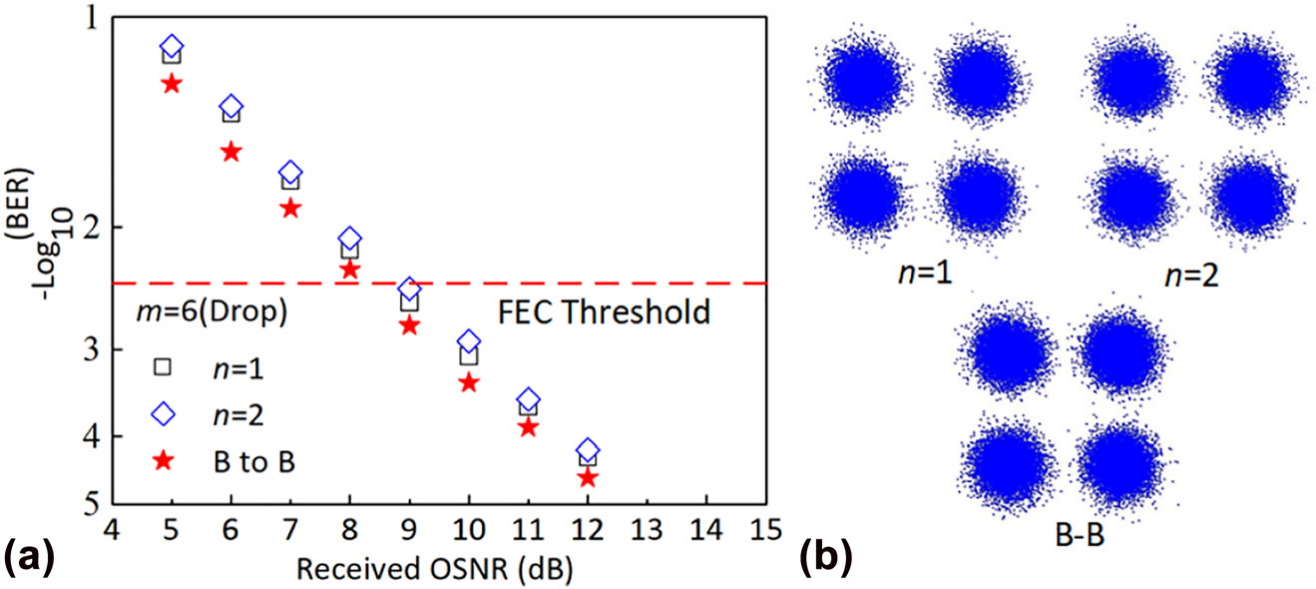
Measured BER performance (a) and the corresponding constellations of QPSK (b) of the WDM dropping transmissions and back-to-back transmission for two adjacent wavelength-channels C_6:1_ and C_6:2_.

Figure 14 shows the measured BER as a function of the received optical signal-to-noise ratio (OSNR) for different channels of *m*th modes and *n*th wavelengths (C_*m:n*_). Here six channels C_6:1_, C_5:2_, C_4:4_, C_3:8_, C_2:12,_ and C_1:18_ are randomly measured and shown in Figure 14(a–f), respectively. Comparing the BER performances of the add/drop transmissions to that of the back-to-back transmission of each channel, the observed OSNR penalties induced by the adding/dropping route of the ROADM are less than 2 dB for all six channels at a BER of 3.8 × 10^−3^ (forward error correction (FEC) threshold). The insets of Figure 14(a–f) show the measured constellations of QPSK signal for the adding and dropping transmission, respectively, showing decent signal quality. WDM transmission performances are further characterized, where two adjacent wavelengths channels C_6:1_, C_6:2_ with a channel spacing of 1.6 nm are used here as an example. As shown in Figure 15(a), the observed OSNR penalties of the dropping transmission at a BER of 3.8 × 10^−3^ for both channels are less than 1 dB because of the low inter-wavelength crosstalk (<−24 dB) of two adjacent wavelength channels of the fabricated ROADM. The constellations of the QPSK signal for C_6:1_, C_6:2_ drop transmission and back-to-back transmission are shown in Figure 15(b). It should be mentioned, that the baud rate of the system is limited by the narrow bandwidth of the MRR switches, which can be improved by adopting higher-order MRRs with box-like responses [61, 62].

Table 3 summarizes the reported silicon ROADMs for WDM, MDM, and hybrid-multiplexed systems. It can be seen that the present ROADM is the first one for hybrid MDM-PDM-WDM systems, working with the largest channel number. On the other hand, the excess loss and the crosstalk increase as the channel number increases. As a result, it is really necessary to develop silicon photonic devices with very high performances, e. g., low-loss waveguide crossings, low-crosstalk PSRs, higher-order MRRs with box-like responses, etc. Furthermore, more efforts should be made to include more functional elements, such as online optical power monitors and variable optical attenuators for channel equalization.

## 4 Conclusions

In summary, we have proposed and demonstrated a silicon-based 96-channel ROADM for hybrid MDM-WDM-PDM systems for the first time. This ROADM consists of a pair of six-channel mode (DE) multiplexers to support six mode channels of dual polarizations, a 6 × 16 array of MRR-based wavelength-selective switches, and 6 × 96 single-mode waveguide crossings for on-chip crossing connection. Such a ROADM integrates more than 1000 elements monolithically, containing 96 MRRs, 576 waveguide crossings, 192 grating couplers, 96 micro-heaters, 112 pads, six PSRs, four asymmetric adiabatic couplers and four asymmetric directional couplers. One can add/drop optical signal to/from any channel carried by any wavelength, any mode and any polarization in the multimode bus waveguide by switching the corresponding MRR-based optical switch. The fabricated ROADM chip was bonded to a PCB for electrical controlling and packaged with a fiber array for light coupling. The ROADM shows on-chip ELs of 5–20 dB, and inter-wavelength crosstalk <−24 dB, and inter-mode crosstalk <−12 dB for adding any one of all 96 channels in the wavelength range of 1560–1590 nm. The system experiments have been demonstrated by using 10-GBaud QPSK signals, showing the observed OSNR penalties induced by the ROADM are less than 2 dB at a BER of 3.8 × 10^−3^. The performance of the proposed ROADM can be further improved by adopting low loss waveguide crossings, and low crosstalk mode MUX/DEMUX. Such hybrid WDM-MDM-PDM ROADM can be extended with more mode channels as well as more wavelength channels for the applications of ultra-high-capacity data routing/switching, which is promising in future optical interconnect networks.
